# Label-free quantitative proteomics of *Corynebacterium pseudotuberculosis* isolates reveals differences between Biovars *ovis* and *equi* strains

**DOI:** 10.1186/s12864-017-3835-y

**Published:** 2017-06-08

**Authors:** Wanderson M. Silva, Edson L. Folador, Siomar C. Soares, Gustavo H. M. F. Souza, Agenor V. Santos, Cassiana S. Sousa, Henrique Figueiredo, Anderson Miyoshi, Yves Le Loir, Artur Silva, Vasco Azevedo

**Affiliations:** 1 Departamento de Biologia Geral, Instituto de Ciências Biológicas, Universidade Federal de Minas Gerais, Belo Horizonte, Minas Gerais Brasil; 2 Instituto de Ciências Biológicas, Universidade Federal do Pará, Belém, Pará Brasil; 3 Centro de Biotecnologia, Universidade Federal da Paraíba, João Pessoa, Paraíba Brasil; 4 Waters Corporation, Waters Technologies Brazil, MS Applications Laboratory, Alphaville, São Paulo, Brasil; 5 INRA, UMR1253 STLO, 35042 Rennes, France; 6 Agrocampus Ouest, UMR1253 STLO, 35042 Rennes, France; 7 Escola de Veterinária, Aquavet, Universidade Federal de Minas Gerais, Belo Horizonte, Minas Gerais Brasil; 8 Departmento de Microbiologia, Imunologia e Parasitologia, Instituto de Ciências Biológicas e Naturais, Universidade Federal do Triângulo Mineiro, Uberaba, Minas Gerais Brasil

**Keywords:** *Corynebacterium pseudotuberculosis*, Caseous lymphadenitis, Ulcerative lymphangitis, Proteomic bacterial, Label-free proteomics, proteomic

## Abstract

**Background:**

*Corynebacterium pseudotuberculosis* is a pathogen classified into two biovars: *C. pseudotuberculosis* biovar *ovis*, the etiologic agent of caseous lymphadenitis and *C. pseudotuberculosis* biovar *equi*, which causes ulcerative lymphangitis. The available whole genome sequences of different *C. pseudotuberculosis* strains have enabled identify difference of genes related both virulence and physiology of each biovar. To evaluate be this difference could reflect at proteomic level and to better understand the shared factors and the exclusive ones of biovar *ovis* and biovar *equi* strains, we applied the label-free quantitative proteomic to characterize the proteome of the strains: 1002_*ovis* and 258_*equi*, isolated from goat (Brazil) and equine (Belgium), respectively.

**Results:**

From this analysis, we characterized a total of 1230 proteins in 1002_*ovi*s and 1220 in 258_*equi* with high confidence. Moreover, the core-proteome between 1002_*ovis* and 258_*equi* obtained here is composed of 1122 proteins involved in different cellular processes, which could be necessary for the free living of *C. pseudotuberculosis*. In addition, 120 proteins from this core-proteome presented change in abundant with statistically significant differences. Considering the exclusive proteome, we detected strain-specific proteins to each strain. When correlated, the exclusive proteome of each strain and proteome with change in abundant, the proteomic differences, between the 1002_*ovis* and 258_*equi*, this related to proteins involved in cellular metabolism, information storage and processing, cellular processes and signaling.

**Conclusions:**

This study reports the first comparative proteomic study of the biovars *ovis* and *equi* of *C. pseudotuberculosis*. The results generated in this study provide information about factors which can contribute to understanding both the physiology and the virulence of this pathogen.

**Electronic supplementary material:**

The online version of this article (doi:10.1186/s12864-017-3835-y) contains supplementary material, which is available to authorized users.

## Background

*Corynebacterium pseudotuberculosis* is a Gram-positive facultative intracellular pathogen of the Corynebacterium, Mycobacterium, Nocardia, and Rhodococcus (CMNR) group. The CMNR group of pathogens has high G + C content in their genomes and shows a specific cell wall organization composed of peptidoglycan, arabinogalactan, and mycolic acids [1]. *C. pseudotuberculosis* is subdivided into two biovars: (i) *C. pseudotuberculosis* biovar *ovis* (nitrate negative) which is the etiologic agent of caseous lymphadenitis in small ruminants [2] and mastitis in dairy cattle [3] and (ii) *C. pseudotuberculosis* biovar *equi* (nitrate positive) that causes ulcerative lymphangitis and abscesses in internal organs of equines [4] and oedematous skin disease in buffalos [5]. *C. pseudotuberculosis* infection is reported worldwide and causes significant economic losses by affecting wool, meat, and milk production [6–9].

Various studies at genome level have been carried out by our research group in order to explore the molecular basis of specific and shared factors among different strains of *C. pseudotuberculosis* that could contribute to such biovar specific pathogenicity. Our studies on whole-genome sequencing and analysis of several *C. pseudotuberculosis* strains belonging to biovar *ovis* and *equi*, isolated from different hosts showed an average genome size of approximately 2,3 Mb, a core-genome having approximately 1504 genes across several *C. pseudotuberculosis* species, and accessory genomes of biovar *equi* and *ovis* composed of 95 and 314 genes, respectively [10–12]. According with pan-genome analysis, *C. pseudotuberculosis* biovar *ovis* presented a more clonal-like behavior, than the *C. pseudotuberculosis* biovar *equi.* In addition, in this *in silico* study was observed a variability most interesting related to pilus genes, where biovar *ovis* strain presented high similarity, while, biovar *equi* strains have a great variability, suggesting that this variability could influence in the adhesion and invasion cellular of each biovar [10].

Apart from the structural genome informatics studies of *C. pseudotuberculosis*, some proteomic studies were conducted to explore the functional genome of this pathogen [13–19]. However, all these proteomic studies were performed using only strains belonging to biovar *ovis*. Until the present time, no proteomic studies were performed between biovar *equi* strains or between biovar *ovis* and biovar *equi* strains. Therefore, to provide insights on shared and exclusive proteins among biovar *ovis* and biovar *equi* strains and to complement the previous studies on functional and structural genomics of *C. pseudotuberculosis* biovars, using LC-MS^E^ approach [13, 18] this study reports for the first time a comparative proteomic analysis of two *C. pseudotuberculosis* strains, 1002_*ovis* and 258_*equi*, isolated from caprine (Brazil) and equine (Belgium), respectively. Our proteomic dataset promoted the validations of previous work* in silico* of *C. pseudotuberculosis*; in addition, the qualitative and quantitative differences in the proteins identified in this present work have potential to help understand the factors that might contribute for pathogenic process of biovar *ovis* and *equi* strains.

## Methods

### Bacterial strain and growth condition

*C. pseudotuberculosis* biovar *ovis* 1002, isolated from a goat in Brazil, and *C. pseudotuberculosis* biovar *equi* 258, isolated from a horse in Belgium, were maintained in brain–heart infusion broth or agar (1.5%) (BHI-HiMedia Laboratories Pvt. Ltd., India) at 37 °C. For proteomic analysis, overnight cultures (three biological replicate to each strain) in BHI were inoculated with a 1:100 dilution in fresh BHI at 37 °C and cells were harvested during the exponential growth at DO_600_ = 0.8 (Additional file 1: Figure S1).

### Protein extraction and preparation of whole bacterial lysates for LC-MS/MS

After bacterial growth, the protein extraction was performed according to Silva et al. [18]. The cultures were centrifuged at 4000 x g at 4 °C for 20 min. The cell pellets were washed in phosphate buffered saline (PBS) and then resuspended in 1 mL of lysis buffer (7 M Urea, 2 M Thiourea, CHAPS 4% and 1 M DTT) and 10 μL of Protease Inhibitor Mix (GE Healthcare, Piscataway, NJ, USA) was added. The cells were broken by sonication at 5 × 1 min cycles on ice and the lysates were centrifuged at 14,000 x g for 30 min at 4 °C. Subsequently, samples were concentrated and lysis buffer was replaced by 50 mM ammonium bicarbonate at pH 8.0 using a 10 kDa ultra-filtration device (Millipore, Ireland). All centrifugation steps were performed at room temperature. Finally the protein concentration was determined by Bradford method [20]. A total of 50 μg proteins from each biological replicate of 1002_*ovis* and 258_*equi* were denatured by using RapiGEST SF [(0.1%) (Waters, Milford, CA, USA)] at 60 °C for 15 min, reduced with DTT [(10 mM) (GE Healthcare)], and alkylated with iodoacetamide [(10 mM) (GE Healthcare)]. For enzymatic digestion, trypsin [(0.5 μg/μL) (Promega, Sequencing Grade Modified Trypsin, Madison, WI, USA)] was added and placed in a thermomixer at 37 °C overnight. The digestion process was stopped by the addition of 10 μL of 5% TFA (Sigma-Aldrich, St. Louis, Missouri, USA) and glycogen phosphorilase (Sigma-Aldrich) was added to the digests to give 20 fmol.uL^−1^ as an internal standard for scouting normalization prior to each replicate injection into label-free quantitation [21].

### LC-HDMS^E^ analysis and data processing

Qualitative and quantitative analysis were performed using 2D RPxRP (two-dimensional reversed phase) nanoUPLC-MS (Nano Ultra Performance Liquid Chromatography Mass Spectrometry) approach with multiplexed Nano Electrospray High Definition Mass Spectrometry (nanoESI-HDMS^E^). To ensure that all samples were injected with the same amount into the columns and to ensure standardized molar values across all conditions, stoichiometric measurements based on scouting runs of the integrated total ion account (TIC) were performed prior to analysis. The experiments were conducted using both a 1 h reversed phase gradient from 7% to 40% (*v*/v) acetonitrile (0.1% *v*/v formic acid) and a 500 nL.min^−1^ on a 2D nanoACQUITY UPLC technology system [22]. A nanoACQUITY UPLC HSS (High Strength Silica) T3 1.8 μm, 75 μm × 15 cm column (pH 3) was used in conjunction with a reverse phase (RP) XBridge BEH130 C18 5 μm 300 μm × 50 mm nanoflow column (pH 10). Typical on-column sample loads were 250 ng of total protein digests for each 5 fractions (250 ng/fraction/load). For all measurements, the mass spectrometer was operated in the resolution mode with a typical m/z resolving power of at least 35,000 FMHW and an ion mobility cell filled with nitrogen gas and a cross-section resolving power at least 40 Ω/ΔΩ. All analyses were performed using nano-electrospray ionization in the positive ion mode nanoESI (+) and a NanoLockSpray (Waters, Manchester, UK) ionization source.

The lock mass channel was sampled every 30 s. The mass spectrometer was calibrated with a MS/MS spectrum of [Glu1]-Fibrinopeptide B human (Glu-Fib) solution (100 fmol.uL^−1^) delivered through the reference sprayer of the NanoLockSpray source.The doubly- charged ion ([M + 2H]^2+^ = 785.8426) was used for initial single-point calibration and MS/MS fragment ions of Glu-Fib were used to obtain the final instrument calibration. Multiplexed data-independent (DIA) scanning with added specificity and selectivity of a non-linear ‘T-wave’ ion mobility (HDMS^E^) experiments were performed with a Synapt G2-S HDMS mass spectrometer (Waters), which was automatically planned to switch between standard MS (3 eV) and elevated collision energies HDMS^E^ (19–45 eV) applied to the transfer ‘T-wave’ CID (collision-induced dissociation) cell with argon gas. The trap collision cell was adjusted for 1 eV, using a mili-seconds scan time previously adjusted based on the linear velocity of the chromatography peak delivered through nanoACQUITY UPLC to get a minimum of 20 scan points for each single peak, both in low energy and at high-energy transmission at an orthogonal acceleration time-of-flight (oa-TOF) from m/z 50 to 2000. The RF offset (MS profile) was adjusted is such a way that the nanoUPLC-HDMS^E^ data are effectively acquired from m/z 400 to 2000, which ensured that any masses observed in the high energy spectra with less than m/z 400 arise from dissociations in the collision cell.

### Database searching and quantification

Following the identification of proteins, the quantitative data were packaged using dedicated algorithms [23, 24] and searching against a database with default parameters to account for ions [25]. The databases used were reversed “on-the fly” during the database queries and appended to the original database to assess the false positive rate (FDR) during identification. For proper spectra processing and database searching conditions, the Protein Lynx Global Server v.2.5.2 (PLGS) with Identity^E^ and Expression^E^ informatics v.2.5.2 (Waters) were used. UniProtKB (release 2013_01) with manually reviewed annotations was used, and the search conditions were based on taxonomy (*Corynebacterium pseudotuberculosis*). We have utilized a database from genome annotation of 1002_*ovis* CP001809.2 version and 258_*equi* CP003540.2 version. These databases were randomized within PLGS v.2.5.2 for generate a concatenated database from both genomes. Thus, the measured MS/MS spectra from proteomic datasets of 1002_*ovis* and 258_*equi* were searched against this concatenated database. The maximum allowed missed cleavages by trypsin were up to one, and variable modifications by carbamidomethyl (C), acetyl N-terminal, phosphoryl (STY) and oxidation (M) were allowed and peptide mass tolerance value of 10 ppm was used [26]. Peptides as source fragments, peptides with a charge state of at least [M + 2H]^2+^ and the absence of decoys were the factors we considered to increase the data quality. The collected proteins were organized by the PLGS Expression^E^ tool algorithm into a statistically significant list that corresponded to higher or lower regulation ratios among the different groups. For protein quantitation, the PLGS v2.5.2 software was used with the IdentityE algorithm using the Hi3 methodology. The search threshold to accept each spectrum was the default value in the program with a false discovery rate value of 4%. The quantitative values were averaged over all samples, and the standard deviations at *p* < 0.05 were determined using the Expression software. Only proteins with a differential expression log2 ratio between the two conditions greater than or equal to 1.2 were considered [26].

### Bioinformatics analysis

The identified proteins in 1002_*ovis* and 258_*equi* were subjected to the bioinformatics analysis using the various prediction tools. SurfG+ v1.0 [27] was used to predict sub-cellular localization, SignalP 4.1.0 server [28] to predict the presence of N-terminal signal peptides for secretory proteins, SecretomeP 2.0 server [29] to identify exported proteins from non-classical systems (positive prediction score greater than to 0.5), LipoP server [30] to determine lipoproteins, Blast2GO [31] and COG database [32] were used for functional annotations. The protein-protein interaction network was generated using Cytoscape version 2.8.3 [33] with a spring-embedded layout.

## Results and discussion

### Characterization of the proteome of *C. pseudotuberculosis* biovar *ovis* and *equi*

**Fig. 1:**
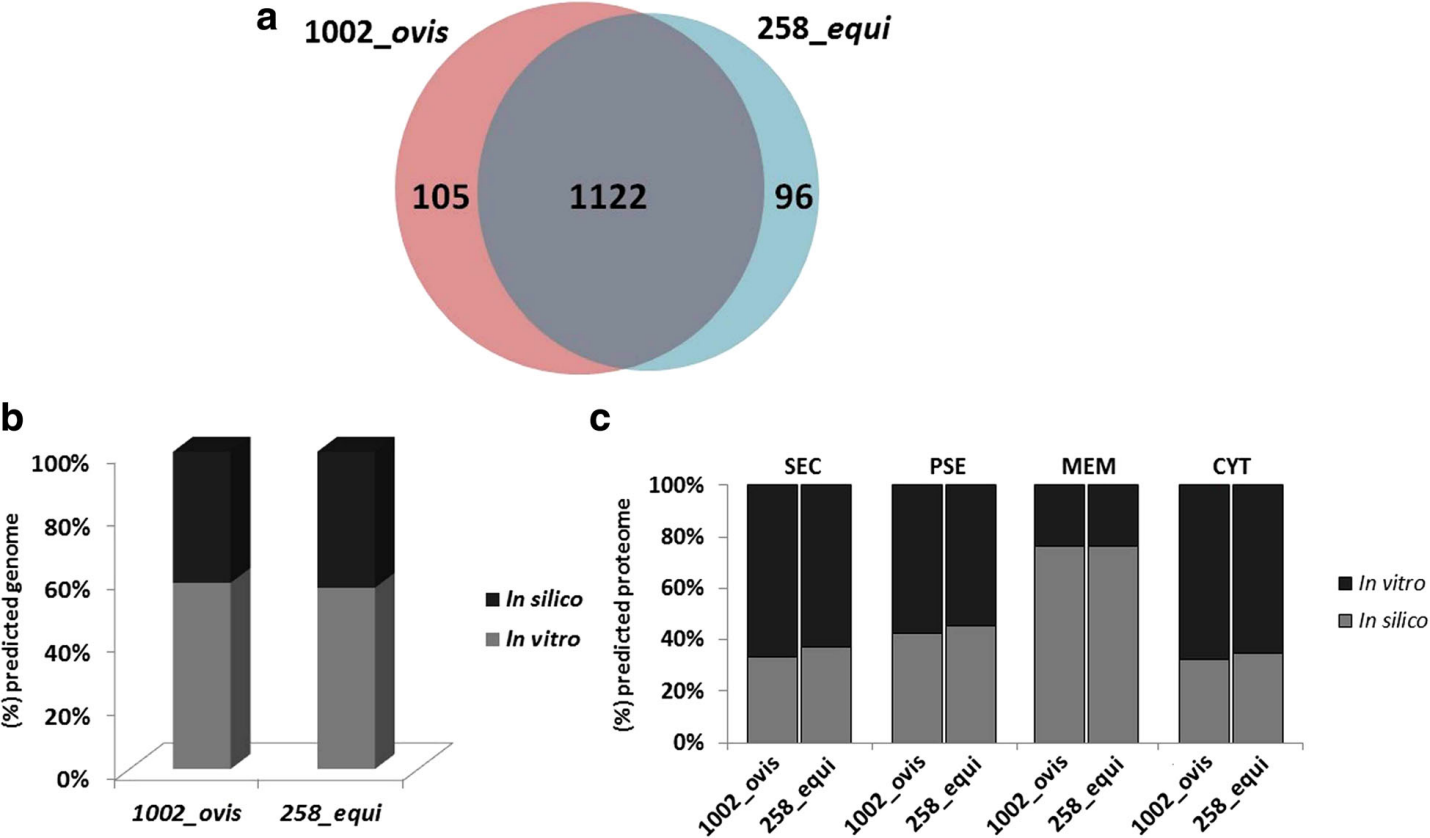
Characterization of the proteome of *C. pseudotuberculosis* and correlation with *in silico* data. **a** Distribution of the proteins identified in the proteome of 1002_*ovis* and 258_*equi*, represented by Venn diagram. **b** Correlation of the proteomic results with *in silico* data of the genomes of 1002_*ovis* and 258_*equi*. **c** Subcellular localization of the identified proteins and correlation with the *in silico* predicted proteome. CYT, cytoplasmic; MEM, membrane; PSE, potentially surface-exposed and SEC, secreted

In this study, we applied the 2D nanoUPLC-HDMS^E^ approach to characterize the proteome of the strains 1002_*ovis* and 258_*equi*. Both strains were grown in BHI media, subsequently proteins were extracted and digested in solution, and then the peptides were analyzed by LC/MS^E^. Our proteomic analysis identified a total of 1227 non-redundant proteins in 1002_*ovis* (Additional file 2: Table S1 and Additional file 3: Table S2) and 1218 in 258_*equi* (Additional file 2: Table S1 and Additional file 4: Table S3) (Fig. 1a). The information about sequence coverage and a number of identified peptides for each protein sequence identified, as well as the information about the native peptide are available at Additional file 5: Table S4 and Additional file 6: Table S5. Altogether from the proteome of these two biovars, we identified a total of 1323 different proteins of *C. pseudotuberculosis* with high confidence (Fig. 1a) and characterized approximately 58% of the predicted proteome of 1002_*ovis* [11] (Fig. 1b). In the case of 258_*equi*, we characterized approximately 57% of the predicted proteome [12] (Fig. 1b). The proteins identified in both proteomes were analyzed by SurfG+ tool [27] to predict the subcellular localization into four categories: cytoplasmic (CYT), membrane (MEM), potentially surface-exposed (PSE) and secreted (SEC) (Fig. 1c). Further, we identified 83% (43 proteins) of the lipoproteins predicted in 1002_*ovis* and 79% (41 proteins) in 258_*equi*. Considering proteins with LPxTG motif which are involved in covalent linkage with peptidoglycan, we identified 6 proteins in 1002_*ovis* and 4 proteins in 258_*equi* that correspond to approximately 38% and 34% of the LPxTG proteins predicted in each strain, respectively.

### The biovar *equi* and biovar *ovis* core proteome

**Fig. 2:**
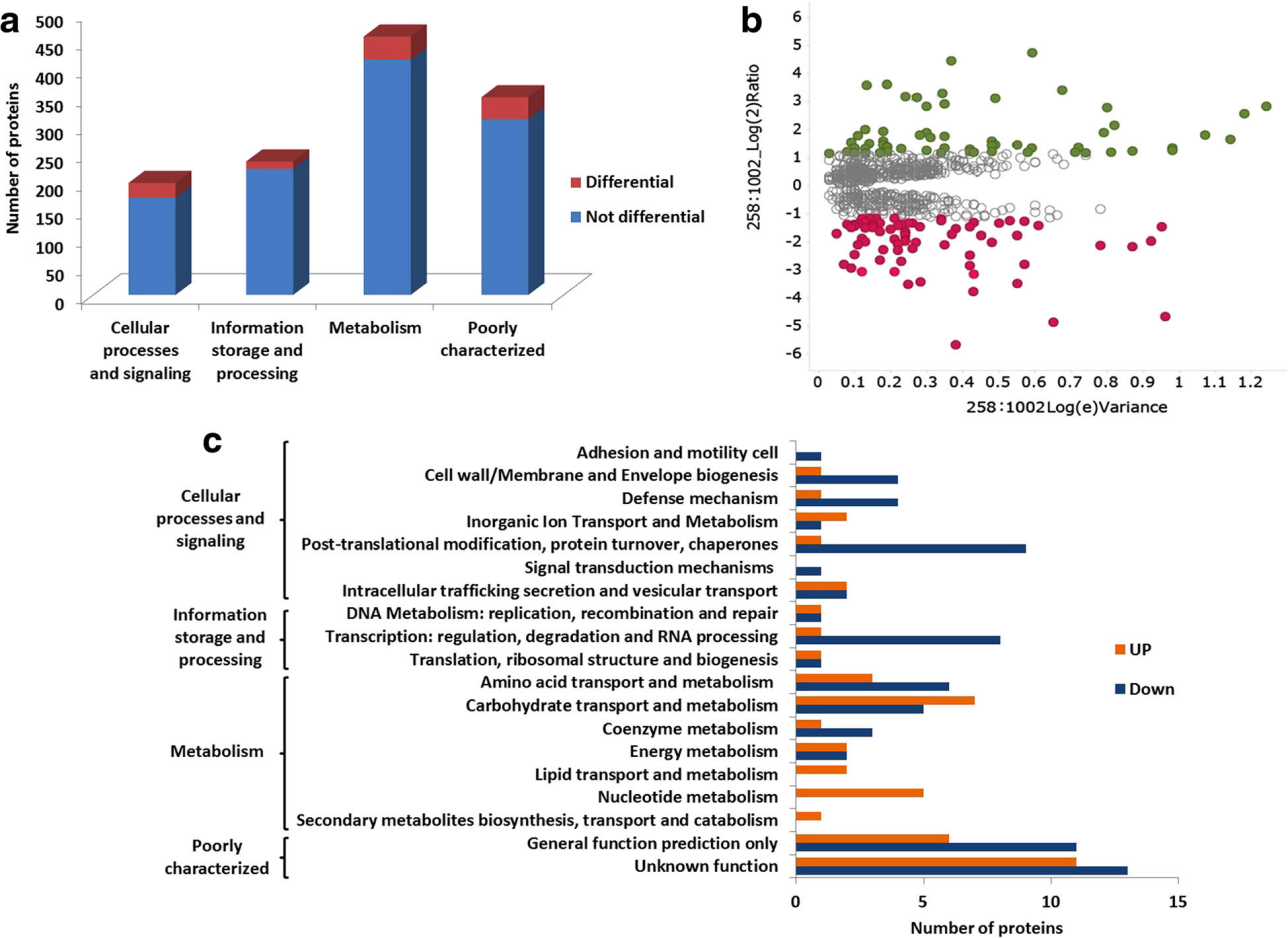
Representative results of the core-proteome 1002_*ovis* and 258_*equi*. **a** Functional distribution of the proteins identified in the core-proteome. **b** Volcano plot generated by differentially expressed proteins, log2 ratio of 258_*equi/*1002_*ovis*. **c** Biological processes differential between 258_*equi* and 1002_*ovis*

The core-proteome, between 258_*equi* and 1002_*ovis* is composed of 1122 proteins (Fig. 1) (Additional file 2: Table S1). Interestingly, when correlated these 1122 proteins with in silico data of the *C. pseudotuberculosis* core-genome [10], we observed that 86% (960 proteins) of the *Open Reading Frame* (ORF) that encodes these proteins are part of the core-genome (Additional file 2: Table S1), what represents approximately 64% of the predicted core-genome of this pathogen. In addition, these data show a set of proteins involved in different cellular processes which could be necessary for the free living of *C. pseudotuberculosis*. The other 14% (262 proteins) of the proteins that constitute the core-proteome are shared by at least one of the 15 strains used in the core-genome study. According to Gene Ontology analysis [31, 32], the 1122 proteins were classified into four important functional groups: (i) metabolism, (ii) information storage and processing, (iii) cellular processes and signaling, and (iv) poorly characterized (Fig. 2a). As observed in the study of *C. pseudotuberculosis* [10] core genome in the categories “metabolism” and “information storage and processing” were detected a large number of proteins.

The label-free quantification was applied to evaluate the relative abundance of the core-proteome of 258_*equi* and 1002_*ovis*. The ProteinLynx Global Server (PLGS) v2.5.2 software with Expression^E^ algorithm tool was used to identify proteins with *p* ≤ 0.05 (Additional file 2: Table S1). Among these proteins, 120 proteins between 258_*equi* and 1002_*ovis* showed difference in level of abundance (log_2_ ratios equal or greater than a factor of 1.2) [26] (Table 1). In this group of proteins that have presented different abundance level (258_*equi*:1002_*ovis*), 49 proteins were more abundant and 71 less abundant (Table 1). To visualize this differential distribution of the core-proteome a volcano plot of the log_2_ ratio of 258_*equi*/1002_*ovis* versus Log (e) Variance was generated (Fig. 2b). Interestingly, the Phospholipase D (Pld), the major virulence factor of *C. pseudotuberculosis*, was more abundant in 258_*equi*, than in 1002_*ovis* (Table 1). The Pld have an important play role in the pathogenic process of *C. pseudotuberculosis*, due to the sphingomyelinase activity of the Pld, this exotoxin increases vascular permeability through the exchange of polar groups attached to membrane-bound lipids and helps the bacteria in spread inside the host [34, 35]. In addition, this exotoxin is able to reduce the viability of both macrophages and neutrophils [34, 36]. In comparative proteomic studies between 1002_*ovis* and C231_*ovis* exoproteome, Pld was detected only in the C231_*ovis* supernatant [13, 15, 16]. A study performed with pld mutant strains presented decreased virulence [37]. Thus, in relation to 258_*equi*, 1002_*ovis* could present a low potential of virulence.

**Table 1:** Differentially regulated proteins between 258_*equi* and 1002_*ovis*

Accession		Description		258:1002 Log(2)Ratio^(a)^	
258_*equi*	1002_*ovis*		Score		*p_*value^(a)^
Cellular processes and signaling					
Adhesion and motility cell					
I3QUW8_CORPS	D9Q5S4_CORP1	Periplasmic zinc binding protein troA	4245,52	-1,32	0
Cell wall/Membrane and Envelope biogenesis					
I3QYI6_CORPS	D9Q3G9_CORP1	Phospho N acetylmuramoyl pentapeptide	166,05	1,22	1
I3QZH4_CORPS	D9Q4F8_CORP1	Corynomycolyl transferase	3886,67	-1,45	0
I3R044_CORPS	D9Q526_CORP1	Peptidoglycan recognition proteino	5283,55	−2,06	0
	D9Q4M0_CORP1	Cell wall channel	2220,85	−2,14	0
I3QYK8_CORPS	D9Q3J1_CORP1	Cell wall peptidase NlpC P60 protein	1207,7	−2,78	0
Defense mechanism					
I3QW82_CORPS	D9Q743_CORP1	Cold shock protein	6171,9	1,37	1
I3R0B7_CORPS	D9Q597_CORP1	DNA protection during starvation protein	70,504,73	−1,48	0
I3QV66_CORPS	D9Q632_CORP1	Cold shock protein	31,703,46	−2458	0
I3QZX1_CORPS	D9Q4V4_CORP1	Protein GrpE	57,665,59	−2,64	0
I3QZW9_CORPS	D9Q4V2_CORP1	Heat shock protein HspR	929,96	−3,43	0
Intracellular trafficking secretion and vesicular transport					
I3QVC7_CORPS	D9Q697_CORP1	ABC type transporter	376,36	2,91	1
I3QZ34_CORPS	D9Q431_CORP1	ABC transporter ATP binding protein	6339,11	1,54	1
I3QZQ5_CORPS	D9Q4N9_CORP1	ABC superfamily ATP binding cassette	25,578,26	−1,38	0
I3R0D8_CORPS	D9Q5B9_CORP1	Oligopeptide transport system permease	705,01	−1,45	0,01
Post-translational modification, protein turnover, chaperones					
I3QWY7_CORPS	D9Q7U6_CORP1	Thioredoxin TrxA	1832,12	3,15	1
I3QVC2_CORPS	D9Q692_CORP1	Thiol disulfide isomerase thioredoxin	157,88	1,80	1
I3QXH3_CORPS	D9Q8C5_CORP1	Proteasome accessory factor PafA2	305,06	−1,32	0
I3QZA4_CORPS	D9Q493_CORP1	Glutaredoxin like protein nrdH	3140,61	−1,34	0
I3QUL7_CORPS	D9Q5I3_CORP1	Peptidyl prolyl cis trans isomerase	49,161,11	−1,44	0
I3QWQ3_CORPS	D9Q7L6_CORP1	Ferredoxin	54,332,67	−1,48	0
I3QW91_CORPS	D9Q753_CORP1	Peptidyl prolyl cis trans isomerase	19,736,36	−1,63	0
I3QV23_CORPS	D9Q5Y2_CORP1	Catalase	52,016,22	−1,70	0
I3QUX7_CORPS	D9Q5T5_CORP1	Glyoxalase Bleomycin resistance protein	18,489,51	−1,99	0
I3QVR1_CORPS	D9Q6M3_CORP1	10 kDa chaperonin	90,387,73	−2,78	0
Signal transduction mechanisms					
I3QY22_CORPS	D9Q8W9_CORP1	Phosphocarrier protein HPr	38,569,08	−2,92	0
Information storage and processing					
DNA Metabolism: replication, recombination and repair					
I3QV41_CORPS	D9Q606_CORP1	Metallophosphoesterase	529,63	1,88	1
I3QWM2_CORPS		Exodeoxyribonuclease 7 small subunit	10,725,45	−2,00	0
Transcription: regulation, degradation and RNA processing					
I3QX71_CORPS	D9Q817_CORP1	SAM dependent methyltransferase y	397,16	1,25	0,96
I3QWD6_CORPS	D9Q7A0_CORP1	TetR family regulatory protein	5685,08	−1,38	0
I3QXS5_CORPS	D9Q8M5_CORP1	N utilization substance protein B homol	16,977,04	−1,42	0
I3QWH0_CORPS	D9Q7D4_CORP1	Transcriptional regulatory protein PvdS	7456,32	−1,48	0
I3QYU0_CORPS	D9Q3T4_CORP1	Ferric uptake regulatory protein	7805,46	−1,76	0
I3QWK3_CORPS	D9Q7G7_CORP1	Transcription elongation factor GreA	77,246,3	−1,87	0
I3QZJ2_CORPS	D9Q4H4_CORP1	Transcriptional regulator	10,476,01	−1,93	0
I3QUZ7_CORPS	D9Q5V6_CORP1	Nucleoid associated protein ybaB	81,447,09	−3,04	0
I3QUU4_CORPS	D9Q5Q1_CORP1	YaaA protein	25,362,05	−3,14	0
Translation, ribosomal structure and biogenesis					
I3R0I2_CORPS	D9Q5F9_CORP1	Ribosomal RNA small subunit methyltrans	395,99	1,29	1
I3QWD9_CORPS	D9Q7A3_CORP1	30S ribosomal protein S14	4756,75	−1,47	0
Metabolism					
Amino acid transport and metabolism					
	D9Q3B4_CORP1	Glutamate dehydrogenase	1534,86	3,56	1
I3QXI1_CORPS	D9Q8D2_CORP1	Aspartate ammonia lyase	2326,21	1,60	1
I3QWF9_CORPS	D9Q7C4_CORP1	Glycine betaine transporter	136,23	1,21	1
I3QV21_CORPS	D9Q5Y0_CORP1	Aspartate semialdehyde dehydrogenase	8778,47	−1,28	0
I3QWZ5_CORPS	D9Q7V4_CORP1	Cysteine desulfurase	1813,31	−1,31	0
I3QXT1_CORPS	D9Q8N1_CORP1	Chorismate synthase	5341,49	−1,41	0
I3QXI5_CORPS	D9Q8D5_CORP1	Phosphoribosyl ATP pyrophosphatase	25,184,13	−1,90	0
I3QXL8_CORPS	D9Q8G8_CORP1	UPF0237 protein Cp258 1096	16,011,21	−1,96	0
I3QZZ5_CORPS	D9Q4X5_CORP1	Urease subunit beta	4349,97	−2,12	0
Carbohydrate transport and metabolism					
I3R0E6_CORPS	D9Q5C6_CORP1	Aldose 1 epimerase	221,55	2,78	1
I3QV93_CORPS	D9Q660_CORP1	Formate acetyltransferase 1	9381,31	2,00	1
I3QZB7_CORPS	D9Q4A5_CORP1	Phosphoglucomutase	359,43	1,77	1
I3QWW1_CORPS	D9Q7S1_CORP1	L lactate permease	103,53	1,38	0,99
	D9Q8W7_CORP1	PTS system fructose specific EIIABC	191,71	1,35	1
I3QY20_CORPS	D9Q8W6_CORP1	1 phosphofructokinase	2879,52	1,32	1
I3R064_CORPS	D9Q545_CORP1	L lactate dehydrogenase	5695,04	1,32	1
I3R051_CORPS	D9Q691_CORP1	Probable phosphoglycerate mutase	2014,4	−1,24	0
	D9Q396_CORP1	PTS system fructose specific IIABC	507,53	−1,25	0
I3QWR8_CORPS	D9Q7N1_CORP1	Sucrose 6 phosphate hydrolase	6075,08	−1,34	0
I3QYN0_CORPS	D9Q3L2_CORP1	Glycine cleavage system H proteino	91,529,52	−1,38	0
I3QWH7_CORPS	D9Q7D8_CORP1	Glyceraldehyde 3 phosphate dehydrogenase	9529,4	−2,68	0
Coenzyme metabolism					
	D9Q862_CORP1	ATP dependent dethiobiotin synthetase B	104,92	−1,24	1
I3QUY1_CORPS	D9Q5T9_CORP1	Pyridoxal biosynthesis lyase PdxSo	1029,15	−1,42	0
I3QXF8_CORPS	D9Q8B1_CORP1	Precorrin 8X methyl mutase	34,981,98	−1,77	0
I3QXE1_CORPS	D9Q893_CORP1	Hemolysin related protein	3609,76	1,35	0
Energy metabolism					
I3QUS5_CORPS	D9Q5N2_CORP1	NADH dehydrogenase	3257,66	3,14	1
I3QZ12_CORPS	D9Q411_CORP1	Malate dehydrogenase	11,220,59	1,22	1
I3QXN4_CORPS	D9Q8I5_CORP1	Cytochrome oxidase assembly protein	297,19	−1,86	0,09
I3QYB6_CORPS	D9Q3A4_CORP1	Nitrogen regulatory protein P II	16,165,93	−2,29	0
Inorganic Ion Transport and Metabolism					
I3R097_CORPS	D9Q575_CORP1	Cation transport protein	882,31	−4,64	1
I3QZG5_CORPS	D9Q4F0_CORP1	Trk system potassium uptake protein trk	2973,22	1,25	1
I3QVU4_CORPS	D9Q6Q3_CORP1	Hemin binding periplasmic protein hmuT	607,64	1,71	1
I3QVT3_CORPS	D9Q6P2_CORP1	Manganese ABC transporter substrate binding	3917,5	1,51	0
Lipid transport and metabolism					
I3QUM7_CORPS	D9Q5J0_CORP1	Phospholipase D	25,847,67	3,27	1
I3QZM9_CORPS	D9Q4L5_CORP1	Secretory lipase	1254	3,08	1
Nucleotide metabolism					
I3QZR5_CORPS	D9Q4P8_CORP1	Purine phosphoribosyltransferase	227,51	4,45	1
I3QZF7_CORPS	D9Q4E2_CORP1	Phosphoribosylformylglycinamidine synth	978,57	1,45	1
I3QXV6_CORPS	D9Q8Q6_CORP1	Adenine phosphoribosyltransferaseo	907,16	1,44	1
I3QZ07_CORPS	D9Q406_CORP1	Nucleoside diphosphate kinase	14,996,33	1,35	1
I3QZG8_CORPS	D9Q4F3_CORP1	HIT family protein	4039,81	1,21	1
Secondary metabolites biosynthesis, transport and catabolism					
I3QWA4_CORPS	D9Q766_CORP1	Multidrug resistance protein norMo	208,76	1,35	0,98
Poorly characterized					
General function prediction only					
I3QXE6_CORPS	D9Q898_CORP1	Methyltransferase type 11	102,29	2,84	0,99
I3QWH3_CORPS	D9Q7D6_CORP1	Enoyl CoA hydratase	225,9	2,14	1
	D9Q5M7_CORP1	Unknown function	2201,88	1,93	1
I3QZJ7_CORPS	D9Q4I2_CORP1	Carbonic anhydrase	550,46	1,81	1
I3QWR6_CORPS	D9Q7M9_CORP1	Unknown function	246,05	1,24	0,98
I3QX38_CORPS		Cutinase	11,245,11	−1,48	0
I3QXY3_CORPS	D9Q8T2_CORP1	Chlorite dismutase	32,986,51	−1,51	0
I3QW56_CORPS	D9Q720_CORP1	Methylmalonyl CoA carboxyltransferase 1	4283,03	−1,52	0
I3QWE6_CORPS	D9Q7B0_CORP1	Serine protease	5615,41	−1,54	0
I3QX14_CORPS	D9Q7X2_CORP1	Aldo keto reductase	6121,73	−1,63	0
I3QWR9_CORPS	D9Q7N2_CORP1	Alpha acetolactate decarboxylase	1839,59	−1,73	0
I3QUU3_CORPS	D9Q5Q0_CORP1	UPF0145 protein Cp258 0101	3922,57	−1,77	0
I3QV73_CORPS	D9Q639_CORP1	Hydrolase domain containing protein	5759,37	−2,23	0
I3QZM7_CORPS	D9Q4L2_CORP1	Rhodanese related sulfurtransferase	7428,74	−2,46	0
I3QYY9_CORPS	D9Q3Y7_CORP1	Ankyrin domain containing proteino	11,948,84	−2,84	0
I3QW24_CORPS	D9Q6Y4_CORP1	Hydrolase domain containing protein	15,589,93	−3,04	0
Unknown function					
I3R0H3_CORPS	D9Q5F1_CORP1	Unknown function	7862,73	3,59	1
I3QX01_CORPS	D9Q7W0_CORP1	Unknown function	276,68	3,43	1
I3QYP3_CORPS	D9Q3P0_CORP1	Unknown function	1609,29	2,84	1
I3QWZ8_CORPS	D9Q7V7_CORP1	Unknown function	1476,56	2,58	1
I3R0H2_CORPS	D9Q5F0_CORP1	Unknown function	572,19	1,90	1
I3QY71_CORPS	D9Q919_CORP1	Unknown function	1466,6	1,78	1
I3QWL3_CORPS	D9Q7H7_CORP1	Unknown function	304,96	1,65	0,98
I3QUW1_CORPS	D9Q5R7_CORP1	Unknown function	504,13	1,60	1
I3QYF2_CORPS	D9Q3D7_CORP1	Unknown function	985,97	1,47	1
I3QWR5_CORPS	D9Q7M8_CORP1	Unknown function	103,03	1,45	1
I3QVG0_CORPS	D9Q6C8_CORP1	Unknown function	4085,83	1,39	1
I3QXI8_CORPS	D9Q8D8_CORP1	Unknown function	87,929,25	−1,35	0
I3QVZ1_CORPS	D9Q6U7_CORP1	Unknown function	64,404,7	−1,38	0
I3R094_CORPS	D9Q572_CORP1	Unknown function	4367,05	−1,68	0
I3QYW4_CORPS	D9Q3V8_CORP1	Unknown function	15,928,9	−1,96	0
I3QUX9_CORPS	D9Q5T7_CORP1	Unknown function	8100,51	−2,00	0
I3QYG8_CORPS	D9Q3F3_CORP1	Unknown function	78,035,52	−2,09	0
I3QUZ5_CORPS	D9Q5V4_CORP1	Unknown function	77,763,68	−2,09	0
I3QUR3_CORPS	D9Q5M1_CORP1	Unknown function	12,731,48	−2,27	0
I3QVV7_CORPS	D9Q6R6_CORP1	Unknown function	8564,11	−3,47	0
I3QXX0_CORPS	D9Q8S0_CORP1	Unknown function	19,485,3	−3,50	0
I3QW02_CORPS	D9Q6W1_CORP1	Unknown function	49,581,23	−3,76	0
I3QVS0_CORPS	D9Q6N1_CORP1	Unknown function	66,162,63	−4,87	0
I3R0G5_CORPS	D9Q5E4_CORP1	Unknown function	39,265,48	−5,65	0

The 120 differential proteins were organized by cluster of orthologous groups, and when evaluated the different biological processes that comprise each category listed above, we observed that 19 process were differentials between 258_*equi* and 1002_*ovis* (Fig. 2c, Additional file 7: Figure S2 and Additional file 8: Figure S3). The majority of the more abundant proteins (258_*equi*:1002_*ovis*) are related to cellular metabolism. On other hand, the majority of the less abundant proteins (258_*equi*:1002_*ovis*) are classified as poorly characterized or of unknown function. However, when proteins of known or predicted function are evaluated the majority of the less abundant proteins are related to cellular processes and signaling.

### Difference among the major functional classes identified from the core-proteome analysis of 1002_*ovis* and 258_*equi*

#### Metabolism

During the infection process, pathogens need to adjust their metabolism in response to nutrient availability inside and outside the host. In our proteomic study, we identified several proteins related to different metabolic pathways. To determine the metabolic network of each strain, the proteins identified in this study were analyzed using Kyoto Encyclopedia of Genes pathways and Genomes (KEGG) [38]. A total of 321 and 320 proteins, corresponding to 1002_*ovis* and 258_*equi* respectively, were mapped onto different metabolic pathways (Additional file 9: Figure S4 and Additional file 10: Figure S5). We observed differences in the metabolism of the biovars, related to Amino acid transport and metabolism, Carbohydrate transport and metabolism, Coenzyme metabolism, Energy metabolism, Lipid transport and metabolism, Nucleotide metabolism and Secondary metabolites biosynthesis, transport and catabolism. Difference in the metabolism cellular, also already were observed in others comparative proteomic study of *C. pseudotuberculosis* [13, 16, 17, 19], as well as in the *Mycobacterium tuberculosis* pathogen [39].

Interestingly, the PTS system fructose-specific EIIABC component (PstF) related to carbohydrate metabolism was more abundant in 258_*equi*, than in 1002_*ovis* (Table 1). This protein showed increased abundance in field isolates of *C. pseudotuberculosis* biovar *ovis* grown in BHI when compared to C231_*ovis*, a reference strain [19]. This increased abundance of PstF in 258_*equi*, suggests that this protein could be important to the transport of carbon source both biovar *ovis* and biovar *equi* strains. On the other hand, the Precorrin 8X methyl mutase involved in cobalamin and vitamin B12 synthesis can be required only in biovar *ovis* strains, this protein beside being more abundant in 1002_*ovis* (Table 1), was also detected with greater abundance in the field isolates of *C. pseudotuberculosis* biovar *ovis* after having been grown in BHI [19]. Glutamate dehydrogenase (GDH) was detected more abundant in 258_*equi* (Table 1). A study performed with the *M. bovis* pathogen showed that GDH contributes to the survival of this pathogen during macrophage infection [40].

In *C. pseudotuberculosis*, it was demonstrated that genes related the iron-acquisition are involved in the virulence of this pathogen [41]. In the core-proteome of 1002_*ovis* and 258_*equi*, we detected proteins involved in this process, like CiuA, FagC and FagD; however, all these proteins were not differentially regulated between the two strains (Additional file 2: Table S1). On the other hand, HmuT protein, related to hemin uptake, was more abundant in 258_*equi* (Table 1). Additionally, we have also detected a cell surface hemin receptor in the exclusive proteome of this strain. Heme represents the major reservoir of iron source for many bacterial pathogens that rely on surface-associated heme-uptake receptors [42]. The HmuT is a lipoprotein that acts as a hemin receptor. The *hmuT *gene is part of the operon *hmuTUV*, an ABC transport system (haemin transport system), which is normally present in pathogenic *Corynebacterium* [43, 44]. In addition, in the pathogen *C. ulcerans*, HmuT is required for normal hemin utilization [44].

### Information storage and processing

Of the total protein of proteins identify in the category “information storage and processing” the majority of the differential proteins were less abundant in 258_*equi* (Table 1). Only, Metallophosphoesterase involved in DNA repair, SAM dependent methyltransferase related to transcriptional process and Ribosomal RNA small subunit methyltransferase I involved in translation process were more induced in 258_*equi*. In 1002_*ovis* the Exodeoxyribonuclease 7 important protein related to the DNA-damage pathway was more induced in this strain. In addition, we identified the TetR family regulatory protein as more abundant in 1002_*ovis*, this result was also observed in field isolates of *C. pseudotuberculosis* from sheep infected naturally [19]. TerR proteins are related to regulation of multidrug efflux pumps, antibiotic biosynthesis, catabolic process and cellular differentiation process [45]. Others important transcriptional regulators also were induced in 1002_*ovis* such as PvdS and GreA regulators.

### Cellular processes and signaling

Our proteomic analyses detected differentially regulated proteins belonging to different antioxidant systems. These could contribute to the survival of C. *pseudotuberculosis* in various stress conditions, such as reactive oxygen species (ROS) and reactive nitrogen species (RNS), which are generally found in macrophage. The three major thiol-dependent antioxidant systems in prokaryotic pathogens are the thioredoxin system (Trx), the glutathione system (GSH-system) and the catalase system [46]. Thioredoxin TrxA and Thiol-disulfide isomerase thioredoxin were more abundant in 258_*equi* (Table 1). These proteins are involved in the Trx-system, which has a major role against oxidative stress [46]. However, proteins like catalase and glutaredoxin (nrdH) were less abundant in 258_*equi* (Table 1), being more active in 1002_*ovis*. Catalase plays an important role in resistance to ROS and RNS, as well as in the virulence of *M. tuberculosis* [47]. The protein NrdH has a glutaredoxin amino acid sequence and thioredoxin activity. It is present in *Escherichia coli* [48] and *C. ammoniagenes* [49], as well as in bacteria where the GSH system is absent, such as *M. tuberculosis* [50]. Thus, the presence of NrdH may represent one more factor that contributes to the resistance of *C. pseudotuberculosis* against ROS and RNS during the infection process, as well as to the maintenance of the balance of intracellular redox potential. Proteins like NorB and Glyoxalase/Bleomycin, which play roles in the nitrosative stress response of 1002_*ovis*, were identified in the exclusive proteome of this strain (Additional file 3: Table S2) [14, 18]. These results shown that beside of present proteins with difference in abundance both strains present a set of proteins that could contribute to adaptive process under stress conditions.

### Difference proteomic observed in the exclusive proteome of 258_*equi* and 1002_*ovis*

We found respectively 105 and 96 proteins in the exclusive proteome of 1002_*ovis* and 258_*equi* (Fig. 1) (Additional file 3: Table S2 and Additional file 4: Table S3), related to different biological process (Additional file 7: Figure S2 and Additional file 8: Figure S3). Interestingly, in this exclusive proteome of 1002_*ovis* and 258_*equi*, we detected specific proteins in each strain (Table 2, Additional file 3: Table S2 and Additional file 4: Table S3). In the exclusive proteome of 258_*equi*, the ORFs that codify twenty proteins are annotated as pseudogene in 1002_*ovis* (Table 2, Additional file 3: Table S2 and Additional file 4: Table S3). On the other hand, the ORFs that encode six proteins were not detected in the genome of 1002_*ovis*. These proteins are two CRISPR, MoeB, and three unknown function proteins. CRISPR is an important bacterial defense system against infections by viruses or plasmids, this immunity is obtained from the integration of short sequences of invasive DNA ‘spacers’ into the CRISPR loci [51].

**Table 2:** Exclusive proteins identified in 258_*equi* and 1002_*ovis*

Locus	Locus	Description	Biological Process
		1002_*ovis*	
Cp1002_1457	−	DNA methylase^b^	DNA Metabolism: replication, recombination and repair
Cp1002_1872	Cp258_1887	Collagen binding surface protein Cna^d^	Adhesion and motility cell
Cp1002_1859	Cp258_1875	Sdr family related adhesin^d^	Adhesion and motility cell
Cp1002_2025	Cp258_2050	Glycoside hydrolase 15 related protein^d^	Carbohydrate transport and metabolism
Cp1002_0387	Cp258_0396	Neuraminidase Sialidase^d^	Lipid transport and metabolism
Cp1002_0262	Cp258_0266	Ppx/GppA phosphatase family^d^	General function prediction only
Cp1002_1151	Cp258_1168	Zinc metallopeptidase^d^	General function prediction only
Cp1002_0077	Cp258_0091	Unknown function^d^	Unknown function
		258_*equi*	
Cp258_0374	−	MoeB protein^a^	Coenzyme metabolism
Cp258_1675	−	CRISPR associated protein^a^	DNA Metabolism: replication, recombination and repair
Cp258_0028	−	CRISPR-associated protein^a^	DNA Metabolism: replication, recombination and repair
Cp258_0076	−	Unknown function^a^	Unknown function
Cp258_0585	−	Unknown function^a^	Unknown function
Cp258_0586	−	Unknown function^a^	Unknown function
Cp258_0896	Cp1002_0888	Acetolactate synthase^c^	Amino acid transport and metabolism
Cp258_0465	Cp1002_0455	Cystathionine gamma synthase^c^	Amino acid transport and metabolism
Cp258_0313	Cp1002_0310	Aminopeptidase G^c^	Amino acid transport and metabolism
Cp258_0893	Cp1002_0884	Dihydroxy acid dehydratase^c^	Amino acid transport and metabolism
Cp258_1223	Cp1002_1203	Inositol 1 monophosphatase^c^	Carbohydrate transport and metabolism
Cp258_1360	Cp1002_1337	Unknown function^c^	Coenzyme metabolism
Cp258_1909	Cp1002_1892	Aldehyde dehydrogenase^c^	Energy metabolism
Cp258_0123	Cp1002_0109	ABC type metal ion transport system^c^	Inorganic Ion Transport and Metabolism
Cp258_1854	Cp1002_1838	Disulfide bond formation protein DsbB^c^	Post-translational modification, protein turnover, chaperones
Cp258_0395	Cp1002_0386	Methionine aminopeptidase^c^	Post-translational modification, protein turnover, chaperones
Cp258_1923	Cp1002_1906	Oligopeptide binding protein oppA^c^	Intracellular trafficking secretion and vesicular transport
Cp258_1549	Cp1002_1541	ABC transporter ATP binding protein^c^	Intracellular trafficking secretion and vesicular transport
Cp258_1566	Cp1002_1561	ABC transporter^c^	Intracellular trafficking secretion and vesicular transport
Cp258_0693	Cp1002_0689	Phosphatase YbjI^c^	General function prediction only
Cp258_1503	Cp1002_1497	Alpha beta hydrolase^c^	General function prediction only
Cp258_1265	Cp1002_1243	Unknown function^c^	General function prediction only
Cp258_0169	Cp1002_0157	NADPH dependent nitro flavin reductase^c^	General function prediction only
Cp258_1351	Cp1002_1329	Unknown function^c^	Unknown function
Cp258_1916	Cp1002_1899	Unknown function^c^	Unknown function
Cp258_2099	Cp1002_2077	Unknown function^c^	Unknown function

(^a^) Strain-specific protein, ORF detected only in the genome of 258*_equi*

(^b^)Strain-specific protein, ORF detected only in the genome of 1002*_ovis*

(^c^) *ORF* predicted like pseudogene in 1002_*ovis*

(^d^) *ORF* predicted like pseudogene in 258_*equi*

The distinction between the biovar *ovis* and biovar *biovar equi strains* is based on a biochemical assay, where biovar ovis strains are negative for nitrate reduction, whereas biovar equi strains are positive [52]. However, to date, there is no available information regarding the molecular basis underlying nitrate reduction in *C. pseudotuberculosis* biovar *equi*. MoeB is involved in the molybdenum cofactor (Moco) biosynthesis, which plays an important role in anaerobic respiration in bacteria and also are required to activation of nitrate reductase (NAR) [53]. In the closely related pathogen *M. tuberculosis* several studies have showed the great importance of molybdenum cofactor in its virulence and pathogenic process, mainly macrophage intracellular environmental [54]. Therefore, more studies are necessary to explore the true role of Moco both physiology and virulence of biovar *equi* strains. Other protein that also could contribute to resistance of 258_*equi* macrophage is NADPH dependent nitro/flavin reductase (NfrA), a pseudogene in 1002_*ovis*. In addition, studies performed in *Bacillus subtilis* showed that NfrA is involved in both oxidative stress [55] and heat shock resistance [56].

In 1002_*ovis*, only the ORF that encodes a DNA methylase was not found in the 258_*equi* genome (Table 2, Additional file 3: Table S2 and Additional file 4: Table S3). In addition, the ORFs that codifies seven proteins identified in the exclusive proteome of the strain 1002_*ovis* are annotated like pseudogene in 258_*equi* (Table 2, Additional file 3: Table S2 and Additional file 4: Table S3). Inside this group, we have identified important proteins involved in the process of adhesion and invasion cellular, which might contribute in the pathogenesis of 1002_*ovis*. Adhesion to host cells is a crucial step that favors the bacterial colonization; this process is mediated by different adhesins [57]. We identified proteins such as: collagen binding surface protein Cna-like and Sdr family related adhesin, which are members of the collagen-binding microbial surface components recognizing adhesive matrix molecules (MSCRAMMs) (Table 2). This class of proteins is present in several Gram positive pathogens and plays an important role in bacterial virulence by acting mainly in the cellular adhesion process [58–61].

Another detected protein that might contribute to the virulence of 1002_*ovis* is Neuraminidase (NanH) (Table 2). This protein belongs to a class of glycosyl hydrolases that contributes to the recognition of sialic acids exposed on host cell surfaces [62]. In *C. diphtheriae*, it was demonstrated that a protein with trans-sialidase activity promotes cellular invasion [63, 64]. In addition, NanH was reported to be immunoreactive in the immunoproteome of 1002_*ovis*, showing the antigenicity of this protein [65]. Interestingly, genomic difference in relation to gene involved in the adhesion and invasion process, also already were observed between biovar *ovis* strain and biovar * equi* strains, mainly in genes related to pilus [10, 12]. According to pathogenic process of each biovar, unlike biovar *equi* strain*s*, which rarely causes visceral lesions [4], biovar *ovis* strains, are responsible mainly by visceral lesions [2, 35], what requires a high ability to adhere and invade the host cell, thus these protein could be responsible by this ability of biovar *ovis* strain in attacks visceral organs.

### Proteogenomic analysis

In our proteomic analysis, the measured MS/MS spectra from the proteomic datasets of 1002_*ovis* and 258_*equi* were searched against a concatenated database composed by genome annotation of 1002_*ovis* CP001809.2 version and 258_*equi* CP003540.2 version for identify possible errors or unannotated genes. Thus, by adopting more stringent criteria of considering only proteins with a minimum representative of two peptides and a FDR < 1%, we identified five proteins in 1002_*ovis* and seven proteins in 258_*equi*, which were not previously annotated. All parameters, as well as, the peptides sequence which were used for identification of these proteins are shown in Additional file 11: Table S6 and Additional file 12: Table S7. The proteins identified in this proteogenomic analysis are associated to different biological processes. For instance, the Aminopeptidase N involved in the amino acid metabolism was detected in 1002_*ovis*, whereas the Cobaltochelatase (cobN), associated to cobalt metabolism, glutamate dehydrogenase (gdh) involved in the L-glutamate metabolism, the PTS system fructose specific EIIABC related to fructose metabolism and the Phosphoribosylglycinamide formyltransferase involved in the purine biosynthesis were all detected in 258_*equi*. Proteins involved in DNA processes, such as Uracil DNA glycosylase in 258_*equi*; and Exodeoxyribonuclease 7 small subunit in 1002_*ovis* were also detected in both strains. Proteins with general function prediction only and unknown function were also identified in both strains.

## Conclusion

In conclusion, we used a label-free quantitative approach to compare, for the first time, the proteome of *C. pseudotuberculosis* strains belonging to both *ovis* and *equi* biovars. Taken together, the findings reported here show a set of shared and exclusive factors of 1002_*ovis* and 258_*equi* at the protein level, which can contribute to understanding both the physiology and the virulence of these strains. In addition, the functional analysis of the genome of 1002_*ovis* and 258_*equi* allows the in silico validation of data of the genome of these strains. Thus, the proteins identified here may be used as potential new targets for the development of vaccines against *ovis* and *equi C. pseudotuberculosis* in future investigations.

### Availability of supporting data

The datasets supporting the results of this article were then concatenated into a *xlsx file at peptide and protein level to fulfill the requirements and is available at supplemental material including sequence coverage and a number of identified peptides for each protein sequence identified. It also includes the native peptide information.

## Additional files

Additional file 1: Figure S1. Growth rates in BHI media of 1002_*ovis* (blue circles) and 258_*equi* (red triangles). (JPEG 278 kb)

Additional file 2: Table S1. Total list of proteins identified in the core-proteome of 1002_*ovis* and 258_*equi*. (XLSX 215 kb)

Additional file 3: Table S2. Total list of proteins identified in the exclusive proteome of 1002_*ovis*. (XLSX 20 kb)

Additional file 4: Table S3. Total list of proteins identified in the exclusive proteome of 258_*equi*. (XLSX 21 kb)

Additional file 5: Table S4. Total list of peptide and proteins identified 1002_*ovis*. (XLSB 31769 kb)

Additional file 6: Table S5. Total list of peptide and proteins identified 258_*equi. (XLSB 33204 kb)*

Additional file 7: Figure S2. The protein-protein interaction network of 1002_*ovis*. (A) General interactome of differentially regulated proteins, identified in the exclusive proteome of 1002_*ovis*. The proteins are marked with different shapes: exclusive proteome, circle; more abundant, square; less abundant, rhombus. The biological processes were marked with different colors: amino acid transport and metabolism, yellow; secondary metabolites biosynthesis, transport and catabolism, aquamarine; inorganic ion transport and metabolism, orange; coenzyme metabolism, brown; carbohydrate transport and metabolism, chartreuse green; nucleotide metabolism, cerulean; energy metabolism, olive; lipid transport and metabolism, viridian; adhesion and motility cell, crimson; iuntracellular trafficking secretion and vesicular transport, persian blue; signal transduction mechanisms, maroon; cell wall/membrane and envelope, gray; defense mechanism, red; post-translational modification, protein turnover, chaperones, electric blue; DNA metabolism, replication, recombination and repair, violet; translation, ribosomal structure and biogenesis, amber; transcription, regulation, degradation and RNA processing, salmon; poorly characterized, white. (JPEG 3310 kb)

Additional file 8: Figure S3. The protein-protein interaction network of 258_*equi*. (A) General interactome of the differentially regulated proteins, identified in the exclusive proteome of 258_*equi*. The proteins are marked with different shapes: exclusive proteome, circle; more abundant, square; less abundant, rhombus. The biological processes are marked with different colors: amino acid transport and metabolism, yellow; secondary metabolites biosynthesis, transport and catabolism, aquamarine; inorganic ion transport and metabolism, orange; coenzyme metabolism, brown; carbohydrate transport and metabolism, chartreuse green; nucleotide metabolism, cerulean; energy metabolism, olive; lipid transport and metabolism, viridian; adhesion and motility cell, crimson; intracellular trafficking secretion and vesicular transport, persian blue; signal transduction mechanisms, maroon; cell wall/membrane and envelope, gray; defense mechanism, red; post-translational modification, protein turnover, chaperones, electric blue; DNA metabolism, replication, recombination and repair, violet; translation, ribosomal structure and biogenesis, amber; transcription, regulation, degradation and RNA processing, salmon; poorly characterized, white. (JPEG 4178 kb)

Additional file 9: Figure S4. Metabolic network of 1002_*ovis*. Red line, proteins identified in the proteomic analysis, other colors represent proteins not identified in this study. (JPEG 8633 kb)

Additional file 10: Figure S5. Metabolic network of 258_*equi*. Red line, proteins identified in the proteomic analysis, other colors represent proteins not identified in this study. (JPEG 1267 kb)

Additional file 11: Table S6. Proteins identified in 1002_*ovis* by Proteogenomics. (XLSX 216 kb)

Additional file 12: Table S7. Proteins identified in 258_*equi* by Proteogenomics. (XLSX 266 kb)

## Abbreviations

2D-RP: Two-dimensional reversed phase
C: Citosine
FDR: False discovery rate
G: Guanine
HSS: High strength silica
LC/MS: Liquid chromatograph mass spectrometry
nanoESI-HDMS: Nano Electrospray High Definition Mass Spectrometry
nanoUPLC: Nano Ultra Performance Liquid Chromatography
nanoUPLC-MS: Nano Ultra Performance Liquid Chromatography Mass Spectrometry
PBS: Phosphate buffered saline
PLGS: Protein Lynx Global Server
RNS: Reactive nitrogen species
ROS: Reactive oxygen species
