# Supplementary figures and images for: Label-free quantitative proteomics of Corynebacterium pseudotuberculosis isolates reveals differences between Biovars ovis and equi strains
Source: BMC Genomics. 2017 Jun 8;18:451. doi: 10.1186/s12864-017-3835-y (PMC5463331; doi:10.1186/s12864-017-3835-y)

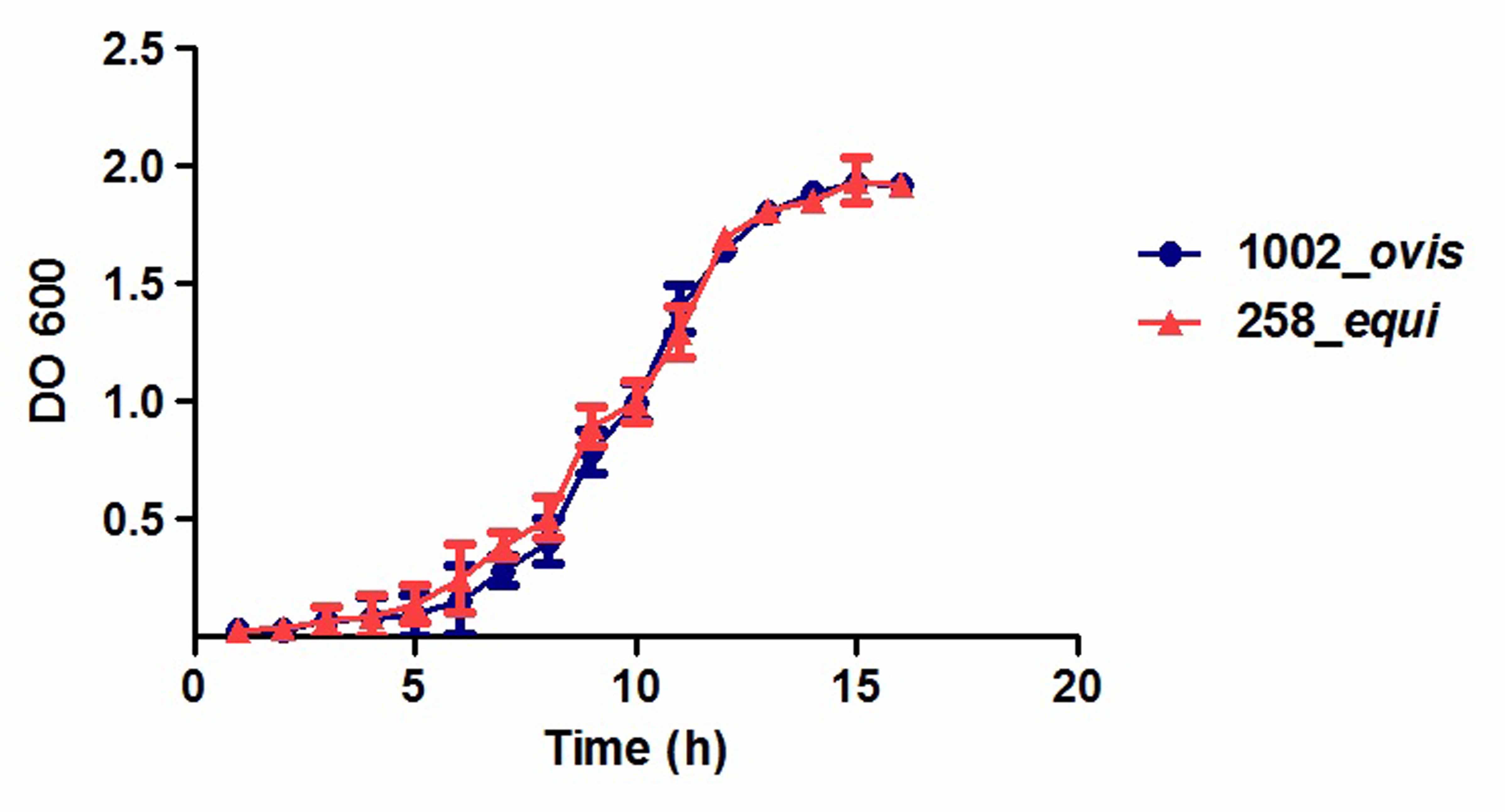

Supplement: Supplementary file 1 — Growth rates in BHI media of 1002_ovis (blue circles) and 258_equi (red triangles). (JPEG 278 kb) [file 12864_2017_3835_MOESM1_ESM.jpg]

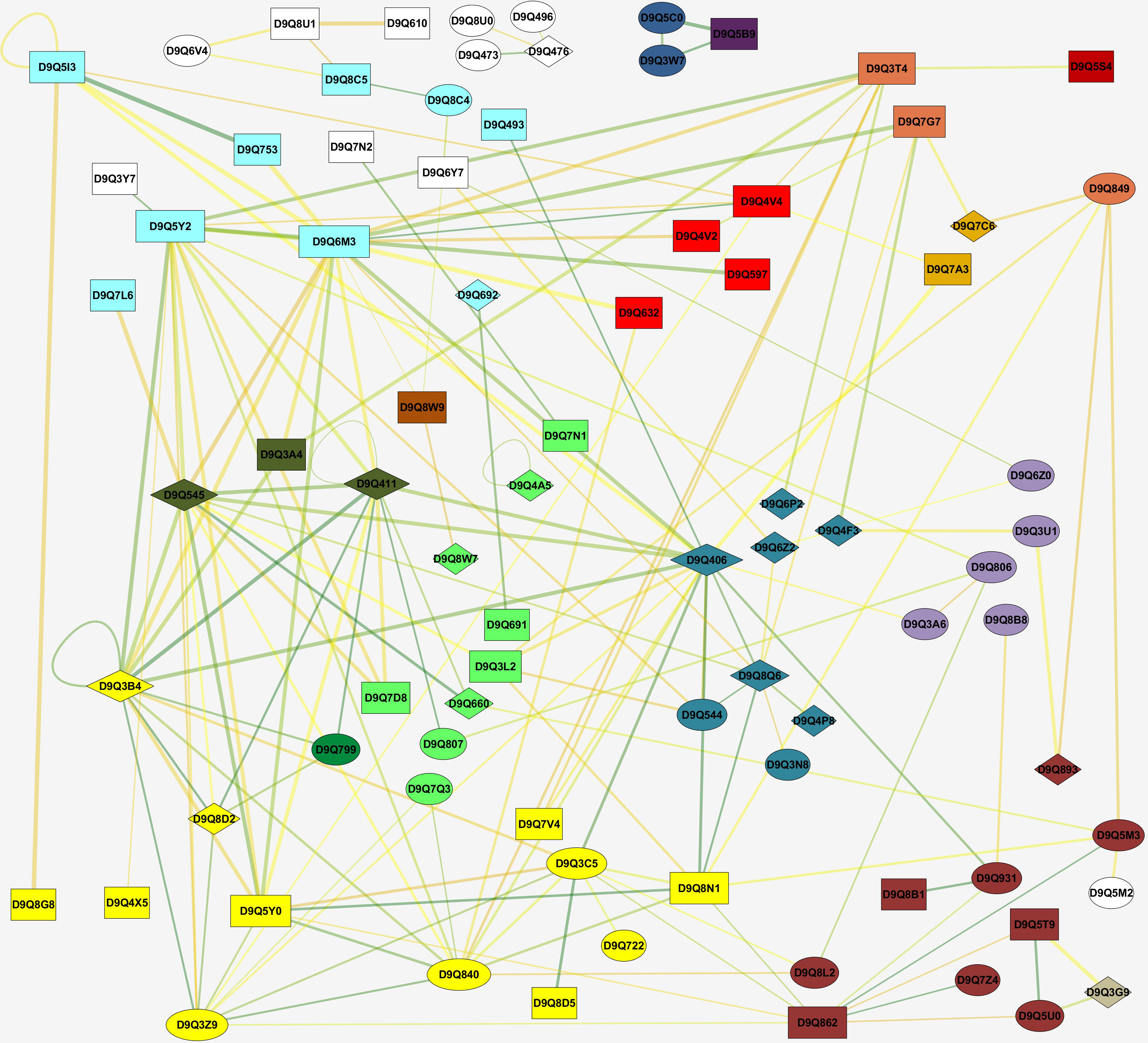

Supplement: Supplementary file 7 — The protein-protein interaction network of 1002_ovis. (A) General interactome of differentially regulated proteins, identified in the exclusive proteome of 1002_ovis. The proteins are marked with different shapes: exclusive proteome, circle; more abundant, square; less abundant, rhombus. The biological processes were marked with different colors: amino acid transport and metabolism, yellow; secondary metabolites biosynthesis, transport and catabolism, aquamarine; inorganic ion transport and metabolism, orange; coenzyme metabolism, brown; carbohydrate transport and metabolism, chartreuse green; nucleotide metabolism, cerulean; energy metabolism, olive; lipid transport and metabolism, viridian; adhesion and motility cell, crimson; iuntracellular trafficking secretion and vesicular transport, persian blue; signal transduction mechanisms, maroon; cell wall/membrane and envelope, gray; defense mechanism, red; post-translational modification, protein turnover, chaperones, electric blue; DNA metabolism, replication, recombination and repair, violet; translation, ribosomal structure and biogenesis, amber; transcription, regulation, degradation and RNA processing, salmon; poorly characterized, white. (JPEG 3310 kb) [file 12864_2017_3835_MOESM7_ESM.jpg]

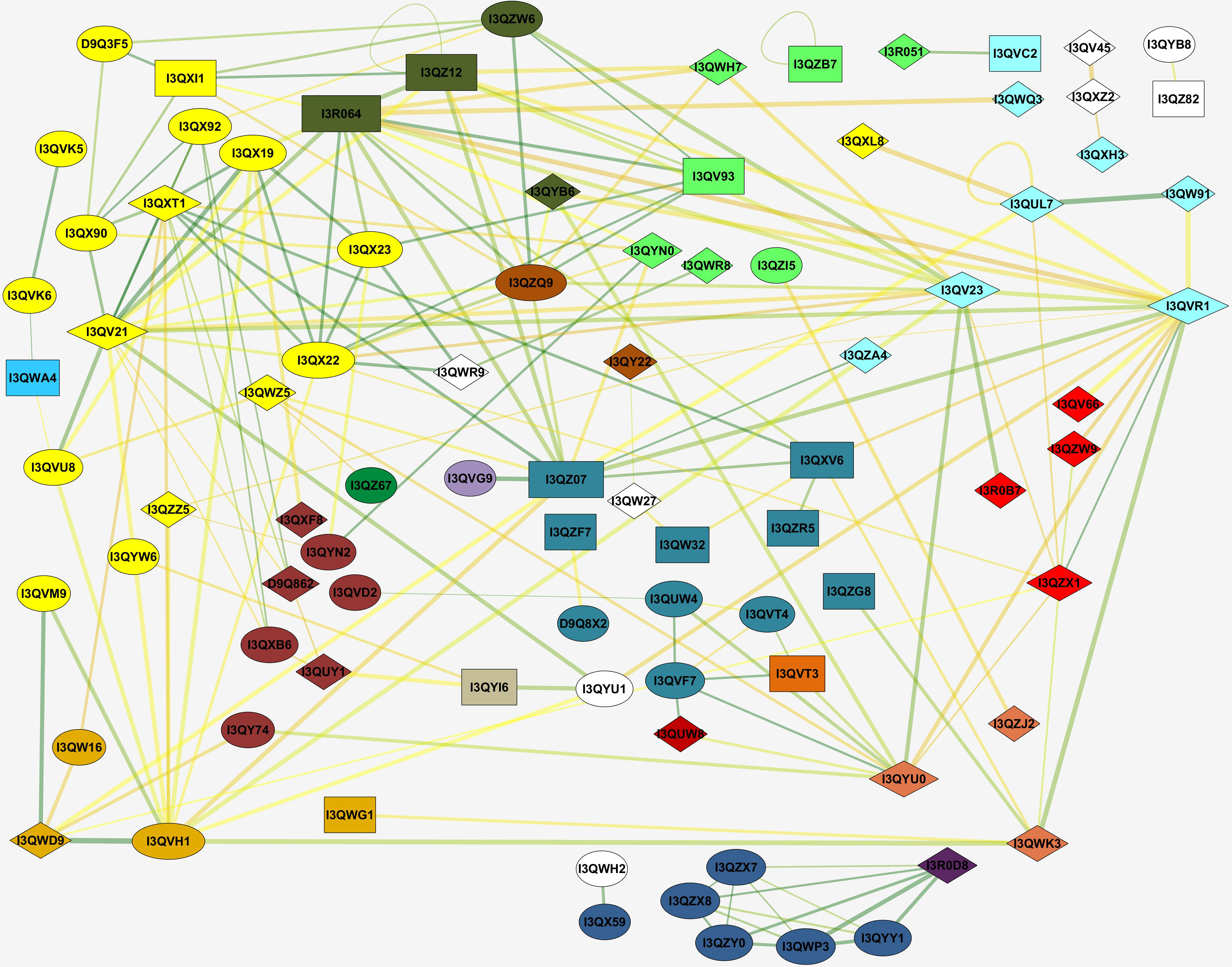

Supplement: Supplementary file 8 — The protein-protein interaction network of 258_equi. (A) General interactome of the differentially regulated proteins, identified in the exclusive proteome of 258_equi. The proteins are marked with different shapes: exclusive proteome, circle; more abundant, square; less abundant, rhombus. The biological processes are marked with different colors: amino acid transport and metabolism, yellow; secondary metabolites biosynthesis, transport and catabolism, aquamarine; inorganic ion transport and metabolism, orange; coenzyme metabolism, brown; carbohydrate transport and metabolism, chartreuse green; nucleotide metabolism, cerulean; energy metabolism, olive; lipid transport and metabolism, viridian; adhesion and motility cell, crimson; intracellular trafficking secretion and vesicular transport, persian blue; signal transduction mechanisms, maroon; cell wall/membrane and envelope, gray; defense mechanism, red; post-translational modification, protein turnover, chaperones, electric blue; DNA metabolism, replication, recombination and repair, violet; translation, ribosomal structure and biogenesis, amber; transcription, regulation, degradation and RNA processing, salmon; poorly characterized, white. (JPEG 4178 kb) [file 12864_2017_3835_MOESM8_ESM.jpg]

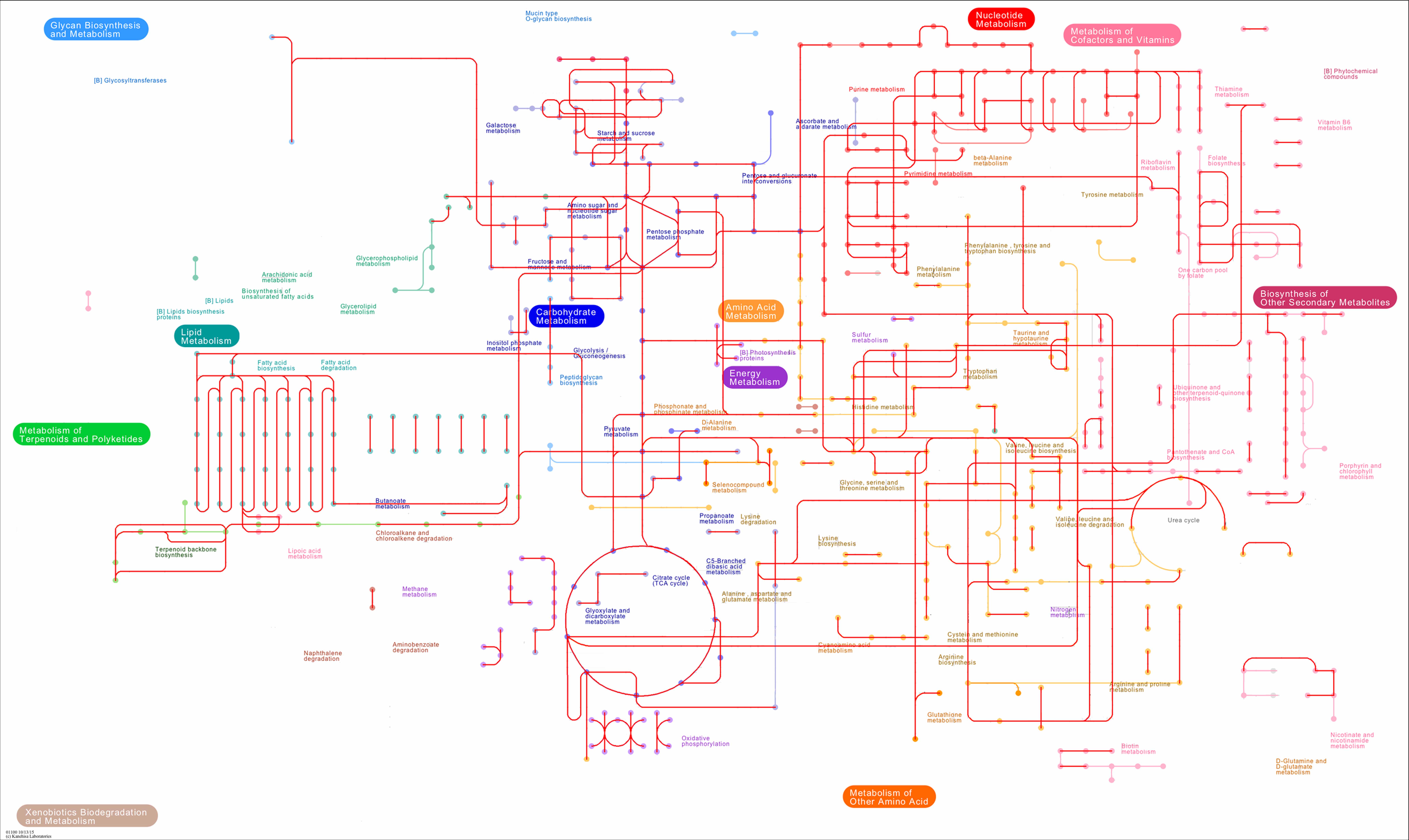

Supplement: Supplementary file 10 — Metabolic network of 258_equi. Red line, proteins identified in the proteomic analysis, other colors represent proteins not identified in this study. (JPEG 1267 kb) [file 12864_2017_3835_MOESM10_ESM.jpg]
